# Supplementary material for: Global constraints within the developmental program of the Drosophila wing
Source: eLife. 2021 Jun 28;10:e66750. doi: 10.7554/eLife.66750 (PMC8257256; doi:10.7554/eLife.66750)
Supplement: Transparent reporting form [file elife-66750-transrepform.docx]

***eLife’s* transparent reporting form**

We encourage authors to provide detailed information *within their submission* to facilitate the interpretation and replication of experiments. Authors can upload supporting documentation to indicate the use of appropriate reporting guidelines for health-related research (see [EQUATOR Network](http://www.equator-network.org/%20)), life science research (see the [BioSharing Information Resource](https://biosharing.org/)), or the [ARRIVE guidelines](http://www.plosbiology.org/article/info:doi/10.1371/journal.pbio.1000412) for reporting work involving animal research. Where applicable, authors should refer to any relevant reporting standards documents in this form.

If you have any questions, please consult our Journal Policies and/or contact us: [editorial@elifesciences.org](mailto:editorial@elifesciences.org).

**Sample-size estimation**

- You should state whether an appropriate sample size was computed when the study was being designed
- You should state the statistical method of sample size computation and any required assumptions
- If no explicit power analysis was used, you should describe how you decided what sample (replicate) size (number) to use

Please outline where this information can be found within the submission (e.g., sections or figure legends), or explain why this information doesn’t apply to your submission:

While our study does include the generation of new data the bulk of the study is a statistical one that builds on a new quantitative strategy to perform statistical morphometrics. We have therefore ensured that our SI and an additional document, titled “Statistical aspects”, gives sufficient explanation and support of our analyses.

We were exploring previously unknown phenomena, and therefore it was unclear what was a good observable, let alone what a sufficient sample size ought to be. In light of this we generated a very large dataset that permits a careful analysis of statistical robustness and significance. As a rule we leverage non-parametric approaches to assessing significance, including bootstrap with replacement and resampling from marginal distributions (also known as shuffling). We pursue a non-parametric approach to avoid any assumptions regarding the generative process of a manifestly very complex phenomena.

We used two sets of wing images. One dataset is publicly available, and it had approximately two hundred images per populations. There were 5 populations in total. The second set of images we produced ourselves we used approximately two hundred images per population. In total we generated 4 populations.

Our analyses, to be found in the additional document “Statistical aspects”, confirms that our sample size is sufficient to generate robust estimation of inferred parameters that we use in our study.

**Replicates**

- You should report how often each experiment was performed
- You should include a definition of biological versus technical replication
- The data obtained should be provided and sufficient information should be provided to indicate the number of independent biological and/or technical replicates
- If you encountered any outliers, you should describe how these were handled
- Criteria for exclusion/inclusion of data should be clearly stated
- High-throughput sequence data should be uploaded before submission, with a private link for reviewers provided (these are available from both GEO and ArrayExpress)

Please outline where this information can be found within the submission (e.g., sections or figure legends), or explain why this information doesn’t apply to your submission:

For details on flies care see Methods Fly Husbandry, Construction of the Outbred Wildtype Population, Temperature and Diet Perturbations, and Wing Mounting.

We imaged all wings without mechanical damage that were sometimes incurred during the mounting process. No other data was disregarded.

**Statistical reporting**

- Statistical analysis methods should be described and justified
- Raw data should be presented in figures whenever informative to do so (typically when N per group is less than 10)
- For each experiment, you should identify the statistical tests used, exact values of N, definitions of center, methods of multiple test correction, and dispersion and precision measures (e.g., mean, median, SD, SEM, confidence intervals; and, for the major substantive results, a measure of effect size (e.g., Pearson's r, Cohen's d)
- Report exact p-values wherever possible alongside the summary statistics and 95% confidence intervals. These should be reported for all key questions and not only when the p-value is less than 0.05.

Please outline where this information can be found within the submission (e.g., sections or figure legends), or explain why this information doesn’t apply to your submission:

There are fundamentally two quantities that form the basis of our analyses of intra and inter population variation. Those being the first and second moments of each genetic and evolutionary population in the phenotype space that we define. It must be stated that these two quantities are provably the most statistically robust features of an empirical data-distribution. Nonlinear analyses that are nowadays common place in even the most standard dimensionality reduction algorithms are more susceptible to noise.

These two quantities go into helping a spectral, or Principle component, analysis of the distributions. The central result of the paper is the striking dominance and importance of the first principle component (eigenvector). It must again be stated that this direction is the most statistically robust direction by construction. We perform a thorough analysis of its robustness in the SI and in an additional document, titled “Statistical aspects”. The only other parameter we infer is the mean phenotype of populations, which is already a quantity that goes into the construction of PCA.

To demonstrate the significance of the PCs that we further analyse and base our conclusions on we adopt a marginal resampling approach. The explicit advantage of this approach over a Marcenko Pasteur (MP) approach is that the closed form analytic solutions for the eigenvalue distributions is based on an assumption of Gaussianity of the data. To avoid this parametric assumption, which is rarely true for real world data, we adopt a marginal resampling approach. This approach gives a spectrum of eigenvalues that could be generated by the nature of the marginal distributions alone, and not due to any covariance in the data. For our data we got at least 70 significant eigenvalues in a representative population.

Robustness in our estimates of mean phenotype is assessed through a straightforward bootstrap with replacement strategy. This is a non-parametric approach to assessing whether the sample size is sufficient to robustly infer a statistical quantity of interest.

Lastly, a very important feature of our analysis was to estimate the direction of variation between the means of two populations. This is the phenotype associated with the difference, say, for example, the phenotypic difference between a genetic mutant set of wings and the WT. To assess whether these directions were robustly estimated you must compare what is measured to a null distributions. Again, in the non-parametric approach embodied in our work, the null distribution is constructed from the data itself. In particular, we compare the measured directions to directions that would emerge by chance were the labels of the different populations of wings to be shuffled. Any alignment between inter-population directions of change and intra-population directions of variance in the shuffled label data is purely due to chance or an artifact of our pipeline In all cases our results are significant relative to this shuffled null. This is explained in detail in the manuscript and SI.

Details of the above analyses can be found in the document titled “Statistical Aspects”.

(For large datasets, or papers with a very large number of statistical tests, you may upload a single table file with tests, Ns, etc., with reference to sections in the manuscript.)

**Group allocation**

- Indicate how samples were allocated into experimental groups (in the case of clinical studies, please specify allocation to treatment method); if randomization was used, please also state if restricted randomization was applied
- Indicate if masking was used during group allocation, data collection and/or data analysis

Please outline where this information can be found within the submission (e.g., sections or figure legends), or explain why this information doesn’t apply to your submission:

We used biological marker as an identifier for a group. Each mutant and sex or a developmental temperature and a type of diet define together with sex define a group.

**Additional data files (“source data”)**

- We encourage you to upload relevant additional data files, such as numerical data that are represented as a graph in a figure, or as a summary table
- Where provided, these should be in the most useful format, and they can be uploaded as “Source data” files linked to a main figure or table
- Include model definition files including the full list of parameters used
- Include code used for data analysis (e.g., R, MatLab)
- Avoid stating that data files are “available upon request”

Please indicate the figures or tables for which source data files have been provided:

Data is available online see references in the paper.
